# Dietary Fluoride Intake and Associated Skeletal and Dental Fluorosis in School Age Children in Rural Ethiopian Rift Valley

**DOI:** 10.3390/ijerph13080756

**Published:** 2016-07-26

**Authors:** Aweke Kebede, Negussie Retta, Cherinet Abuye, Susan J. Whiting, Melkitu Kassaw, Tesfaye Zeru, Masresha Tessema, Marian Kjellevold

**Affiliations:** 1 Food Science and Nutrition Research Directorate, Ethiopian Public Health Institute, P.O. Box 1242, Addis Ababa, Ethiopia; kmelkitu@yahoo.com (M.K.); Nubiya21@gmail.com (T.Z.); masresha88@gmail.com (M.T.); 2 Addis Ababa University, P.O. Box 1176, Addis Ababa, Ethiopia; negussie.retta@gmail.com; 3 ENGINE/Save the Children, P.O. Box 387, Addis Ababa, Ethiopia; cherinet.abuye@savethechildren.org; 4 College of Pharmacy and Nutrition, University of Saskatchewan, Saskatoon, SK S7N 2Z4, Canada; susan.whiting@usask.ca; 5 National Institute of Nutrition and Seafood Research (NIFES), P.O. Box 185, Bergen N-5002, Norway; Marian.Kjellevold@nifes.no

**Keywords:** dietary fluoride, dental fluorosis, skeletal fluorosis, Ethiopian Rift Valley, calcium

## Abstract

An observational study was conducted to determine dietary fluoride intake, diet, and prevalence of dental and skeletal fluorosis of school age children in three fluorosis endemic districts of the Ethiopian Rift Valley having similar concentrations of fluoride (F) in drinking water (~5 mg F/L). The duplicate plate method was used to collect foods consumed by children over 24 h from 20 households in each community (*n* = 60) and the foods, along with water and beverages, were analyzed for fluoride (F) content. Prevalence of dental and skeletal fluorosis was determined using presence of clinical symptoms in children (*n* = 220). Daily dietary fluoride intake was at or above tolerable upper intake level (UL) of 10 mg F/day and the dietary sources (water, prepared food and beverages) all contributed to the daily fluoride burden. Urinary fluoride in children from Fentale and Adamitulu was almost twice (>5 mg/L) the concentration found in urine from children from Alaba, where rain water harvesting was most common. Severe and moderate dental fluorosis was found in Alaba and Adamitulu, the highest severity and prevalence being in the latter district where staple foods were lowest in calcium. Children in all three areas showed evidence of both skeletal and non-skeletal fluorosis. Our data support the hypothesis that intake of calcium rich foods in addition to using rain water for household consumption and preparation of food, may help in reducing risk of fluorosis in Ethiopia, but prospective studies are needed.

## 1. Introduction

Fluoride enters the human body mainly through water and food. Studies show that water is epidemiologically the most important source of dietary fluoride (75%–90% of daily intake) in most areas [1]. Other authors have indicated that considerable exposure risk is also associated with the consumption of fish bones, canned meat, vegetables, grains, local salt (magadi), drinks (especially tea), and prepared food [2,3,4,5]. In some communities, total dietary intake of fluoride from prepared food has been found to be higher than from water [6]. Fluoride intake has usually been calculated indirectly, based on the reported intake of food and drinks consumed using the fluoride content of the most frequently consumed types of food and beverages [7,8]. The method of assessing fluoride intake based on fluoride content in selected food ingredients usually does not take into account the fluoride level of water used for preparing the dish when that aspect is included in the analyses. The Duplicate Plate method therefore can be a better method for assessing total fluoride intake. This method consists of collecting an identical portion of all food and beverages consumed by a subject over 24 h and then analysing the resulting homogenate to determine the fluoride content [5,6].

Absorbed fluoride is rapidly distributed throughout the body, where it is incorporated into calcified tissue (teeth and bones) due to its high affinity for calcium, with virtually no storage in soft tissues [9]. Ninety-nine percent of the total fluoride content of the body is concentrated in calcified tissue. Fluoride is distributed to all tissues via the plasma [10]. Body fluid and soft tissue fluoride concentrations are not under homeostatic control and reflect the recent intake [11]. In bone, the substitution of fluoride for hydroxyl groups in apatite forms fluoroapatite, altering the mineral structure of the bone. Fluoroapatite is more regular and less acid soluble than the compound apatite [12,13]. This increase in bone fluoride content is accompanied by an increase in bone strength up to a certain level, after which bone strength starts decreasing [14,15]. The presence of excessive quantities of fluoride in drinking water and food is accompanied by a characteristic sequence of changes in teeth, bone and periarticular tissues, and is known as fluorosis. Skeletal fluorosis can lead to variable degrees of locomotor disability, ranging from simple mechanical back pain to more severe, and crippling locomotor impairment [16]. Dental fluorosis can lead to eventual loss of tooth surface attrition and pitting [16]. There are other health issues from fluorosis such as neurological impairment [16].

Dental and skeletal fluorosis are important clinical and public health problems in several parts of the world, especially in the Great Rift Valley that extends from northern Syria to central Mozambique in East Africa. The global prevalence of any fluorosis-related health problem has been reported to be about 32% [17] and is endemic in about 22 countries around the world [16]. The severity of either dental or skeletal fluorosis depends on several factors associated with fluoride dose, length of exposure, altitude, and individual differences including health and nutritional status [18,19]. Finally, foods rich in calcium, magnesium and antioxidants are known to reduce fluoride bioavailability and/or ameliorate fluorosis progression [20]. We have shown recently in an animal model that calcium-magnesium salts or a plant source of these minerals effectively reduced apparent fluoride absorption indicating diet may ameliorate fluorosis development [20].

In Ethiopia, data on the level of dietary fluoride intake among endemic populations are scarce, especially in children. A small study (*n* = 28) of children 2–5 years old in Ethiopia [5] found that prepared food contributed mean intakes of 3.5 mg/d and 12.0 mg/d fluoride in two villages with mean fluoride concentrations of 2 mg/L and 14 mg/L in the drinking water, respectively. Therefore the objective of this study was to add to the knowledge of F intakes of children in Ethiopia by determining total dietary fluoride intakes and associated fluorosis prevalences. Dental and skeletal fluorosis were assessed functionally via dental examination and evaluation of movement disorders such as stiffness, as well as some non-skeletal indicators, among a number of school age children in three selected areas of rural Ethiopian Rift Valley varying in diet, use of rain water, and in temperature. These areas had measured water fluoride levels of ≥4.9 mg/L [21].

## 2. Materials and Methods

### 2.1. Study Areas

The study areas were selected based on dietary habits and fluoride content in community water source. In the present study three different districts in Ethiopia were selected: (1) dairy-based-Fentale in North Eastern Oromiya which is pastoral; (2) maize and wheat-based in Adamitulu in Central Oromiya; and (3) maize and finger millet-based in Alaba in the Southern Nations, Nationalities, and Peoples’ Region (SNNPR). While the three areas had similar fluoride content in drinking water of ~5 mg/L, there were differences in mean annual rainfall. Residents in Alaba used rain water while the other two districts were dependent on well water. 

### 2.2. Ethical Clearance

The study was conducted in accordance with the Helsinki Declaration. The study was approved by Ethiopian Public Health Institute (EPHI) (formerly the Ethiopian Health and Nutrition Research Institute) Scientific and Ethical Review Office (SERO) (project identification code: EHNRI/SERO/120/2002). All household heads and care-givers provided their informed consent for inclusion before participation in the study. The households were fully reimbursed for all food items that were collected for each subject.

### 2.3. Households

Households were selected using the following inclusion criteria: the household using the water source typical of the region; residents had lived in the area for more than 10 years and had a child in the age range of 10–15 years who had consumed the water source all of his or her life. Overall 216 households were eligible. All were recruited for the study, with no refusals or dropouts. Information regarding participating households and community were collected using a structured questionnaire that was read aloud to participants. The household questionnaire focused on socio-demographic, knowledge, attitude and practices on defluoridation and fluorosis mitigation. Dietary habits were gathered using a Food Frequency Questionnaire (FFQ) [22]. 

### 2.4. Diet Analysis Using 24-h Duplicate Plate

Two 24-h duplicate portions of all foods and beverages consumed by school age children in the study sites were collected from 20% of households interviewed in Alaba and Adamitulu (chosen randomly) but all households in Fentale (*n* = 20) who had not moved with the others who had gone to higher pasture areas. The estimated amount of food left over from amount served was reduced from the duplicate plate by the care-giver before study personnel weighed and collected the foods into a plastic bag. Foods were collected on one week-day and one weekend day. As the selected dietary areas were Muslim, the inhabitants had no special days of dietary change, in contrast to Ethiopian Orthodox Christians who practice abstention from animal products several days each week. All foods used daily were labelled with unique codes and kept in ice boxes until brought to the laboratory at the Ethiopia Public Health Institute, Addis Ababa. The pooled 24-h foods were homogenized and analyzed in duplicate for fluoride concentration. Separately, all duplicate portion beverages, including coffee (containing about 50% milk in Fentale only), and water consumed by the subjects, were collected and then analyzed for fluoride concentration. In order to calculate fluoride intake as mg per kg (body weight) per day, the body weights of the children were measured by study personnel.

### 2.5. Urine Sample Collection

A spot sample of first morning urine (≥10 mL) was collected by the primary caregiver in the home in a capped plastic tube from each child in each of the 20 households per site. The sample was kept in a cooled ice box until brought to laboratory for fluoride analysis. The ice box was cooled with ice packs which were exchanged every 12 h to prevent the samples from decomposition. Whenever possible the specimens were refrigerated at a nearby health centre or hospital. The samples were labelled with a unique code. In households where there was more than one child, the child was selected randomly.

### 2.6. Chemical Analyses

Samples were analyzed for fluoride (F) using a combination fluoride ion selective electrode (PerfectION) coupled with bench top ion-meter (model 3345, Jenway, London, UK). The methods used were that of Orion for urine and water, and the method of Malde et al. [23] for food. Certified Reference Materials (CRM) were analyzed along the samples to check the accuracy and sensitivity of the fluoride method: “Fluoride in vegetation” (SRM-2695) National Institute of Standards and Technology (NIST, Gaithersburg, MD, USA) and fish tissue prepared in-house by National Institute of Nutrition and Seafood Research (NIFS, Bergen, Norway) was used to validate the method for fluoride analysis.

### 2.7. Dental, Skeletal and Nonskeletal Indicators of Fluorosis

One eligible school age child in the 216 households of the three districts was assessed for dental fluorosis by one trained examiner who held a BSc in dentistry and was familiar with the area, in June 2013 using the Dean Index [24]. Skeletal, periarticular and non-skeletal fluorosis prevalences were determined using clinical symptoms and physical exercise indicators as developed by Susheela et al. [25] and by Shashi and Bhardwaj [26]. We call these indicators of skeletal fluorosis, however the resulting stiffness resulting from fluorosis cannot be easily distinguished as being due to bone (“skeletal”) or joints (“periarticular”) when using these functional indicators of skeletal fluorosis.

## 3. Results

### 3.1. Food Consumption of the Selected Communities

Mean food consumption was higher in Alaba than Fentale and Adamitulu (Table 1), but water and coffee consumption was higher for Fentale than the other two districts. Generally, the ambient outdoor temperature in Fentale (26–38 °C) is highest compared to Alaba (24–29 °C) or Adamitulu (22–28 °C) [27]. We noted that fluid consumption was highest in the children from Fentale. Coffee was consumed with a large proportion of milk in Fentale which appeared to have reduced ingested fluoride and fluorosis by reducing the intake of fluoride contaminated water. The temperature in the Rift Valley generally decreases during June–September due to rainfall [27]. The fluid intake in Fentale was higher than Adamitulu and Alaba. The mean fluid intake correlated with the altitude and mean annual temperature of these three districts. 

### 3.2. Total Fluoride Intake

Table 1 shows high F levels in the water in all three districts. These water levels also translate into high food and beverage levels, as foods and beverages are prepared locally using local water sources. Effectively, total F intake was double that of water alone, but some differences between districts was observed. The dietary fluoride contribution of foods in Alaba and Adamitulu, were higher than that of water, while in Fentale the contribution of water was higher. For two of the three districts, intakes were close to 12 mg F per day. When calculated per body weight (BW), F intake was well above the EFSA [28] criteria of having an F intake less than 0.1 mg/kg BW per day. 

In Table 2, distribution of F intakes are shown according to Institute of Medicine (IOM) categories [29] of adequacy and excess. Between 40% and 75% of children were in the range characterized as “tolerable” but 25% to 60% were above the upper level (UL) of 10 mg F per day. The highest percentages exceeding the UL were found in Fentale and Alaba. 

### 3.3. Urinary Fluoride Excretion 

Data of Ektrand and Ehmebo [30] show that fluoride absorption is 100% on fasting stomach and 60% with Ca-rich meal. When we considered food and beverage sources of F, we compared what would be the bioavailable fluoride intake (i.e., 60% of fluoride intake) to urinary concentration in each district and found dietary F was not related to urinary excretion (Table 3). In particular, urinary F of Adamitulu children was much higher than diet. 

### 3.4. Fluorosis Assessment of Children

Upon dental examination, few children showed normal teeth using criteria of Dean on the two most affected teeth [24] (Table 4). Severe and moderate dental fluorosis was found in Alaba and Adamitulu, the highest being in the latter, but none was seen in Fentale. The drinking water fluoride level was similar among the three districts.

Table 5 shows results of determining the prevalence of clinical symptoms of skeletal and non-skeletal fluorosis in the children. The results indicate that children in Alaba showed stiffness of joints at prevalences of 12%–14% with accompanying pain in 4%–9%, but few gastrointestinal (GI) symptoms. Children in Fentale showed 0%–5% prevalences of stiffness, 5% muscle weakness, thirst (5%) but no GI symptoms. Adamitulu showed little prevalence of stiffness but the highest GI symptoms (1%–4%). 

## 4. Discussion

The situation of fluorosis in children living in the Rift Valley of Ethiopia, as described in this study, shows the need for attention to this problem. Our data show children to be at high risk due to excessive F intakes. These came not only from water but also from foods grown and prepared in the region, as well as beverages made with local water. The total F burden is high. In two of the three districts, over half of the children had intakes that exceeded the UL of 10 mg per day. If only water F intakes were considered, the total F intakes would have been underestimated. This is in accordance with previous studies from Ethiopia [5,6]. The high intake of total fluoride and its subsequent absorption was corroborated by urinary F concentration which revealed levels of F in the urine that were indicative of high intake.

We attempted to find an association of urinary F concentration by district based on dietary staple (low or high in calcium) and by use of different water sources (well vs. rain). Urinary fluoride is used as a biomarker for ingested and circulatory fluoride, but the level depends on several factors such as level of exposure, duration of exposure, age, individual response, weight, degree of physical activity and nutrient and water intake [30,31]. The total fluid intake observed was below what is recommended as total beverage intake by the Institute of Medicine as 1.8–2.4 L for boys and 1.6–1.8 L for girls 9–13 and 14–18 years respectively [32] which may be due to the season of sample collection. Timing of urine collection and amount of urine collected might also determine levels. We collected only morning urine and observed that urinary fluoride concentration in Fentale and Adamitulu was very high while that of Alaba was less than half of the others. Fluoride content of the body is not under physiological control and the kidney is the predominant excretion route for the ingested fluoride. However, urinary excretion depends on several factors including age, amount and duration of exposure. According to EFSA [28], retention in bone can be as high as 90% of the absorbed amount in infants, whereas in adults it is about 50% or less.

The three study sites had different characteristics which may explain the differences in total intake as well as in extent of fluorosis. In Adamitulu, the prevalence of intake of F per day above the UL was the lowest, at 25% of the children measured. Although this daily intake level was lower than the other two districts, the dental fluorosis prevalence was higher. According to Murray [33] high fluid intake in hot tropical areas increases the exposure to fluoride and consequently to fluorosis risk. Studies show the development of dental fluorosis at F concentration as low as 0.8 mg/L because of high fluid consumption in hot climatic areas [31]. Given that the total dietary fluoride intakes of at least half the children were above the upper tolerable level of 10 mg/day [29] and 0.1 mg/BW/day [28], and that the mean annual temperature in the area could induce high water consumption [26], it is not surprising that there were clinical indications of fluorosis. Severe and moderate dental fluorosis and possible symptoms of skeletal fluorosis such as stiffness of joints, tingling sensation in the extremities, stiffness in the neck movement and muscle weakness were found in all the study areas. Some mitigation of fluorosis severity was anticipated due to dietary practice of calcium rich foods and anti-oxidant rich fruits and vegetables [20]; however, dietary diversity of the communities was poor, despite potential for intakes from dairy in Fentale or millet in Alaba. 

Excessive intake of fluoride during enamel maturation before tooth eruption from birth to eight years of age (when enamel formation is complete) can lead to reduced mineral content of enamel and to dental fluorosis of permanent teeth. The incidence and severity of dental fluorosis is dose-dependent [24,29]. A study conducted in Ethiopian children by Wondwossen et al. [34] showed that the second molars were the tooth type most frequently affected by dental fluorosis, i.e., would be seen in older children. These authors [34] found the overall prevalence of dental fluorosis (scoring at or above “very mild”) in 12 to 15 years old Ethiopian children consuming drinking water of 0.3–2.2 mg/L fluoride level was 92%. In the three districts, we found prevalence to be similar in two of the districts (Alaba and Adamitulu, ~93%) but lower in Fentale (70%). There was a high rate of severe and moderate dental fluorosis in Alaba and Adamitulu. Although the drinking water fluoride level was similar and annual mean temperature was higher in Fentale, dental fluorosis was lower than those two other districts. Fentale is a milk-drinking community. The differences in dental fluorosis rate of Alaba and Adamitulu might be explained with differences in type of cereals used as the staple food, and the use of rain water. Alaba communities are dependent on finger millet which is a good source of calcium, and maize while Adamitulu residents depend more on maize and wheat, the latter foods being low in calcium. Moreover, rain water harvesting was common in Alaba while there was none in Adamitulu.

The prevalence of skeletal fluorosis can be estimated using clinical symptoms and ability to do physical exercises as developed by Susheela et al. [25] and used by Shashi and Bhardwaj [26]. Our data show that prevalence of clinical symptoms in arms, lower back bone and neck was lower in children than would be observed in adults. However, the findings do support that skeletal fluorosis was present in children at a young age (10–15 years) which is concerning. The other non-skeletal adverse effects of fluoride we saw included complaints of abdominal pain, constipation, intermittent diarrhoea, a bloated feeling, loss of appetite, and a feeling of nausea. Sashi and Bhardwaj [26] in their assessment of non-skeletal fluorosis in endemic areas found muscle weakness (38%), loss of appetite (44%), feelings of nausea (31%), abdominal pain (24%), polydipsia (27%) and polyuria (29%) in adults. Earlier studies have indicated that the incidence and severity of chronic fluoride intoxication are greatly influenced by socioeconomic status, climatic and nutritional status [25].

Limitations to this study include using symptoms for skeletal fluorosis that are not specific; having only spot urine to estimate excretion; sampling at only one point in time for intakes and urinary excretion. The water intake as well as the nature of the food would be expected to vary through the year, and fluorosis signs and symptoms including dental fluorosis would develop after accumulated F burden. However, a strength was a strength was using multiple measurements including duplicate diet, total intake measurement of F rather than just water, collection for urinary F, and both dental and skeletal signs and symptoms. While F intakes and urinary F concentration were indicative of a high F burden, but we were not able to conclude whether dietary patterns in each district, use of well or rain water, temperature or other factors were responsible for differences in fluorosis severity among the districts. Having only urine F concentration from a spot urine meant we had to make only a crude estimation of F excretion. Strengths of this study include having dietary F, urinary F, and fluorosis determinations all three study sites, with urine concentration and intake being done on the same subjects. Although there has been research on this problem by us and others [3,4,5,6,23,34,35] there remains poor knowledge, attitude and practices toward fluoride as a health problem in Ethiopia [21]. For example, the health extension workers are not teaching about excess fluoride and related health consequences. In the affected areas, dental fluorosis is known to start at an early age, but it is perceived as a cosmetic problem in adolescents without acknowledgement of subsequent skeletal and non-skeletal signs, symptoms and health problems. Even dental fluorosis has enormous health consequences in Ethiopia, with older people having teeth so weak and fragile, they have difficulty in chewing hard foods like unfermented dry flat bread, sugar cane and toasted grains.

## 5. Conclusions

High intakes of fluoride by children in three districts in Ethiopia showed 25% to 60% of the children ingesting above the upper level of 10 mg per day, the value set by the IOM to prevent fluorosis [29]. The total F intake was from not only water and beverages but also foods. High intakes were confirmed by high urinary excretion concentrations. In two districts, moderate and severe dental fluorosis was seen in over half of the children examined. Skeletal and non-skeletal signs and symptoms were also reported. In setting national standards and evaluating the possible health consequences of exposure to fluoride, intake from all sources is crucial. Further research is needed into developing strategies in addition to the use of defluoridation technologies, dietary practice of calcium and magnesium rich cereals, vegetables, dairy products and the use of rain water for household consumption and preparation of food might help in reducing risk of fluorosis.

## Figures and Tables

**Table 1:** Characteristics of subjects, dietary intakes of fluoride, and derivation of total dietary fluoride (F) intake (mean ± SD) by children.

Measure	Unit	Fentale	Alaba	Adamitulu
		*n* = 20	*n* = 20	*n* = 20
Age	y	12.4 ± 1.1	11.6 ± 0.9	11.4 ± 1.3
Sex	M, F	9, 11	7, 13	12, 8
Body Weight	kg	32.1 ± 8.3	35.5 ± 2.6	26.6 ± 6.0
Intakes				
Food	g	364 ± 82	905 ± 269	499 ± 183
Water	mL	938 ± 443	736 ± 277	530 ± 177
Beverage **^#^**	mL	551 ± 222	233 ± 112	151 ± 49
F Sources				
Food	F, mg	2.4 ± 1.2	7.9 ± 5.0	4.4 ± 2.2
Water	F, mg	6.5 ± 3.1	4.6 ± 1.7	2.6 ± 0.8
Beverage **^#^**	F, mg	2.3 ± 1.8	0.4 ± 0.4	0.8 ± 0.3
F Intake	Total dietary, mg/day	11.2 ± 2.4	12.9 ± 3.8	7.8 ± 1.8
	Total dietary, mg/BW	0.35 ± 0.1 **^a^**	0.36 ± 0.2 **^a^**	0.29 ± 0.1 **^b^**

BW = body weight; **^#^** Beverage consumed is entirely coffee except in Fentale with added milk; **^a,b^** Cells with different superscript letters are significantly different (*p* < 0.05) by one-way ANOVA.

**Table 2:** Prevalence (%) of daily dietary F intake of children according to IOM classifications for evaluation of F intake levels.

Category ^#^	Fentale	Alaba	Adamitulu	Total
(mg F per day)	(*n* = 20)	(*n* = 20)	(*n* = 20)	(*n* = 60)
0–2 (Adequate intake level)	0.0	0.0	0.0	0.0
2.1–10 (Tolerable level)	45.0	40.0	75.0	53.3
>10 (Above Upper Level)	55.0	60.0	25.0	46.7

**^#^** IOM categories [29].

**Table 3:** Fluoride intake at 60% bioavailability and urinary fluoride concentration.

Fluoride Intake and Excretion by Children	Study Community (*n* = 20 per Site)	Mean ± SD
Bioavailable F **^#^** (mg F per day)	Fentale	6.7 ± 3.1
	Alaba	7.7 ± 4.9
	Adamitulu	4.7 ± 2.7
Urinary fluoride (mg/L)	Fentale	8.4 ± 5.8
	Alaba	3.3 ± 2.2
	Adamitulu	8.9 ± 4.5

**^#^** F intake calculated as 60% bioavailable (Ektrant and Ehmebo [30]).

**Table 4:** Prevalence (%) of dental fluorosis among school age children.

Dental Fluorosis Category ^#^	Fentale	Alaba	Adamitulu	Difference among Districts
	(*n* = 20)	(*n* = 100)	(*n* = 96)	
	%			
Normal	10.0	4.0	4.2	*p* = 0.003
Questionable	20.0	3.0	2.1	
Very Mild: opaque white areas covering 25% of the tooth surface	45.0	33.0	12.5	
Mild: white areas covering 25%–50% of the tooth surface	25.0	40.0	31.3	
Moderate: all surfaces affected, with some brown spots and marked wear on surfaces subject to attrition	0.0	17.0	39.6	
Severe: widespread brown stains and pitting	0.0	3.0	10.4	

**^#^** Assessment was done by a single trained examiner using criteria of Dean et al. [24]. Statistical analysis was done by one-way ANOVA.

**Table 5:** Prevalence of skeletal and non-skeletal symptoms of school age children.

Skeletal and Non-Skeletal Symptoms ^#^	Fentale	Alaba	Adamitulu
	(*n* = 20)	(*n* = 96)	(*n* = 100)
	%		
Cannot bend body and touch floor or toe	0.0 **^a^**	12.0 **^b^**	0.0 **^a^**
Cannot touch chest with chin	5.0 **^a^**	12.0 **^b^**	1.0 **^c^**
Cannot stretch and fold arms to touch back of head	0.0 **^a^**	14.0 **^b^**	0.0 **^a^**
Feel lower back pain	10.0 **^a^**	9.0 **^a^**	11.5 **^a^**
Feel leg pain, joints	10.0 **^a^**	4.0 **^b^**	4.2 **^b^**
Feel arm pain, joints	5.0 **^a^**	4.0 **^a^**	5.2 **^a^**
Feel tingling sensation	5.0 **^a^**	5.0 **^a^**	4.2 **^a^**
Feel neck pain with movement	5.0 **^a^**	5.0 **^a^**	4.2 **^a^**
Feel muscle weakness	5.0 **^a^**	2.0 **^a^**	0.0 **^a^**
Feel loss of appetite	0.0 **^a^**	0.0 **^a^**	2.1 **^a^**
Have nausea	0.0 **^a^**	0.0 **^a^**	1.0 **^a^**
Have abdominal pain	0.0 **^a^**	1.0 **^a^**	4.2 **^a^**
Have bloating	0.0 **^a^**	1.0 **^a^**	2.1 **^a^**
Experience polydipsia (excessive thirst)	5.0 **^a^**	0.0 **^b^**	2.1 **^b^**
Experience polyuria (excess urine volume)	0.0 **^a^**	0.0 **^a^**	0.0 **^a^**
Experience constipation	0.0 **^a^**	3.0 **^a^**	10.4 **^b^**

**^a,b,c^** Different superscript letters indicate significant differences among districts by one-way ANOVA; ^#^ Based on Susheela et al. [25] and Shashi and Bhardwaj [26].
